# Protocol for quantifying drug sensitivity in 3D patient-derived ovarian cancer models

**DOI:** 10.1016/j.xpro.2024.103274

**Published:** 2024-08-21

**Authors:** Kathrin B. Labrosse, Flavio C. Lombardo, Natalie Rimmer, Mónica Núñez López, André Fedier, Viola Heinzelmann-Schwarz, Ricardo Coelho, Francis Jacob

**Affiliations:** 1 Ovarian Cancer Research, Department of Biomedicine, University Hospital Basel and University of Basel, 4031 Basel, Switzerland; 2 Hospital for Women, University Hospital Basel, 4031 Basel, Switzerland

**Keywords:** Cancer, Cell culture, Single Cell

## Abstract

Three-dimensional (3D) *ex vivo* cultures allow the study of cancer progression and drug resistance mechanisms. Here, we present a protocol for measuring on-target drug sensitivity in a scaffold-free 3D culture system through quantification of apoptotic tumor cells. We provide detailed steps for sample processing, immunofluorescence staining, semi-high-throughput confocal imaging, and imaged-based quantification of 3D cultures. This protocol is versatile and can be applied in principle to any patient-derived material.

## Highlights

• 3D *ex vivo* cultures for on-target drug sensitivity testing at single-cell resolution
• Confocal semi-high-throughput imaging and semi-automated image analysis
• Data generated within a 4-week window, essential for personalized oncology settings

## Abstract (editor highlights)

Publisher’s note: Undertaking any experimental protocol requires adherence to local institutional guidelines for laboratory safety and ethics.

## Graphical abstract

**Figure:**
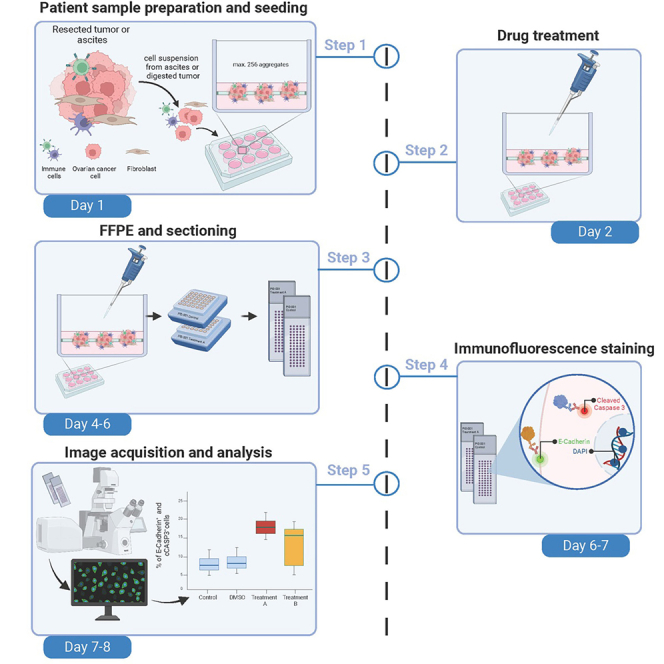

## Before you begin

Epithelial ovarian cancer (EOC) remains a challenging disease, with most of the patients being diagnosed at an advanced stage and having very limited treatment options.^1^ Given these circumstances, there is an imperative need for the exploration of novel therapeutic approaches and personalized treatment plans. Traditional cell culture models (2D) are limited in their ability to capture the complex interactions and the critical microenvironment that plays a crucial role in EOC.^2^ On the contrary the introduction of three-dimensional (3D) *ex vivo* cultures has emerged as a transformative tool, providing a platform that mimics more accurately the *in vivo* situation for studying the EOC complexities.^3^^,^^4^ Notably, 3D cultures allow the recreation of the cell-cell interactions, and response to clinically applied therapy options within a controlled laboratory setting. Taking advantage of a 3D culture system, our protocol offers a method to evaluate on-target drug sensitivity at single-cell resolution in EOC by combining immunofluorescence staining with semi high-throughput confocal microscopy connected to semi-automated image analysis. With our protocol, you have the ability to revisit the paraffin-embedded sections and explore additional research questions through alternative stainings at a later time point. The entire protocol is expected to be completed in approximately two weeks (10 working days in total), meeting the 4-week turnaround time requirement for drug screening technologies designed for personalized oncology approaches.^5^^,^^6^

### Institutional permissions

Prior to working with patient material, approval of the local Institutional Research Board or Ethics Committee is usually required, and patient consent needs to be obtained before study inclusion. For our study, all patient-derived samples and associated data fully adhere to ethical standards. Ethical approval was granted by the Ethical Committee of Nordwest and Zentralschweiz, Switzerland (EKNZ, BASEC ID: 2023-00988, ID: 2017-01900) guaranteeing that all research procedures involving human subjects were conducted under established ethical guidelines.

### Preparation 1: Agarose 3D Petri Dish micro-chips

**Timing: 2 h (10 chips)**

This step involves preparing agarose 3D Petri Dish micro-chips under sterile conditions to ensure they are leak-free. These chips act as a stable platform for *ex vivo* culturing, creating a controlled environment crucial for conducting subsequent experimental procedures such as drug testing and cellular analysis.

**Figure 1:**
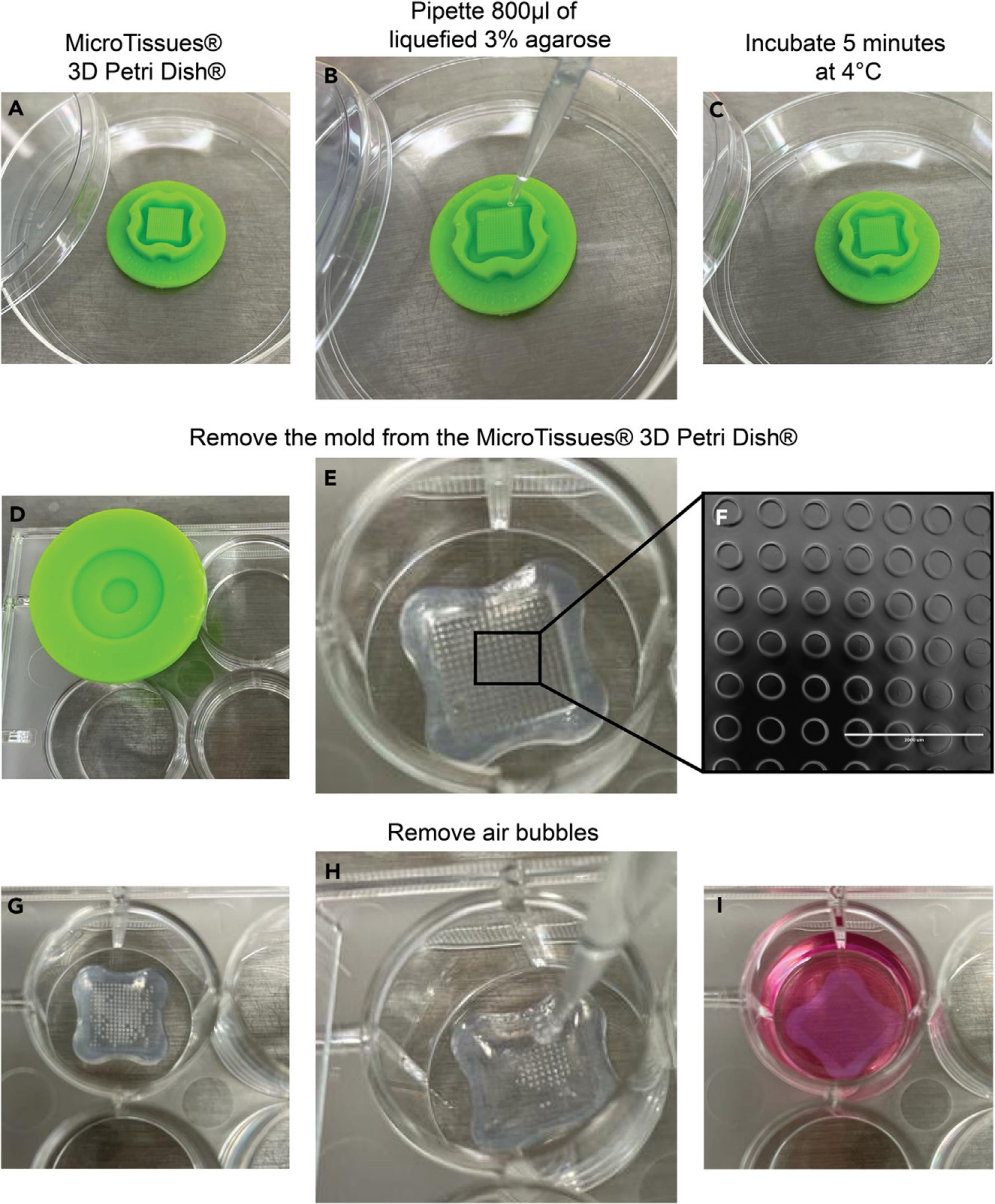
Agarose 3D Petri Dish micro-chip preparation Autoclaved MicroTissues 3D Petri Dish (A). Pipette 800 μL of the liquefied agarose onto MicroTissues 3D Petri Dish (B). Allow the agarose 3D Petri Dish chip to solidify for 5–10 min at 4°C (C). Remove the agarose chip gently from the MicroTissues 3D Petri Dish (D) and place it into one of the wells of a 12-well plate (E). Check under the microscope if all micro-wells are intact (F), add 200 μL of PBS, and check for air bubbles (G). Remove air bubbles by carefully pipetting the PBS up and down (H). Add 2000 μL of Ovarian TumorMACS medium to each well of the 12-well plate (I).
  c. Let the agarose 3D Petri Dish micro-chip solidify for 5–10 min at 4°C.
    ***Note:*** The mold containing the agarose should be placed inside a petri dish for sterility purposes (Figure 1C).
  d. Gently push out the chip in one of the wells of a 12-well plate as exemplified in Figures 1D–1F.
  e. Add 200 μL of PBS onto the chip. After 1 min, check if the 3D Petri Dish micro-chip is leaking:
    i. If yes, discard the chip and start over.
    ii. If not, add 2 mL of PBS to the well containing the 3D Petri Dish micro-chip and remove air bubbles by carefully pipetting up and down (Figures 1G and 1H), after remove the air bubbles remove the PBS and add 2 mL of Ovarian TumorMACS medium (Figure 2I).
      **CRITICAL:** This is a delicate step because the agarose chips are fragile. Carefully loosen the chip all around with various movements before you pop it out completely.

1. Prepare agarose 3D Petri Dish micro-chips.
  a. Heat the pre-autoclaved agarose 3% (w/v) in the microwave at 600 W in cycles of 30 s, until the agarose is completely melted.
    ***Note:*** Ensure that the lid of the glass bottle containing the agarose solution is partially open.
  b. Carefully pipet 800 μL of liquified 3% agarose onto the MicroTissues 3D Petri Dish micro-mold spheroids, avoiding formation of bubbles (Figures 1A and 1B).
    ***Note:*** Onwards, the steps are performed under sterile conditions.

**Figure 2:**
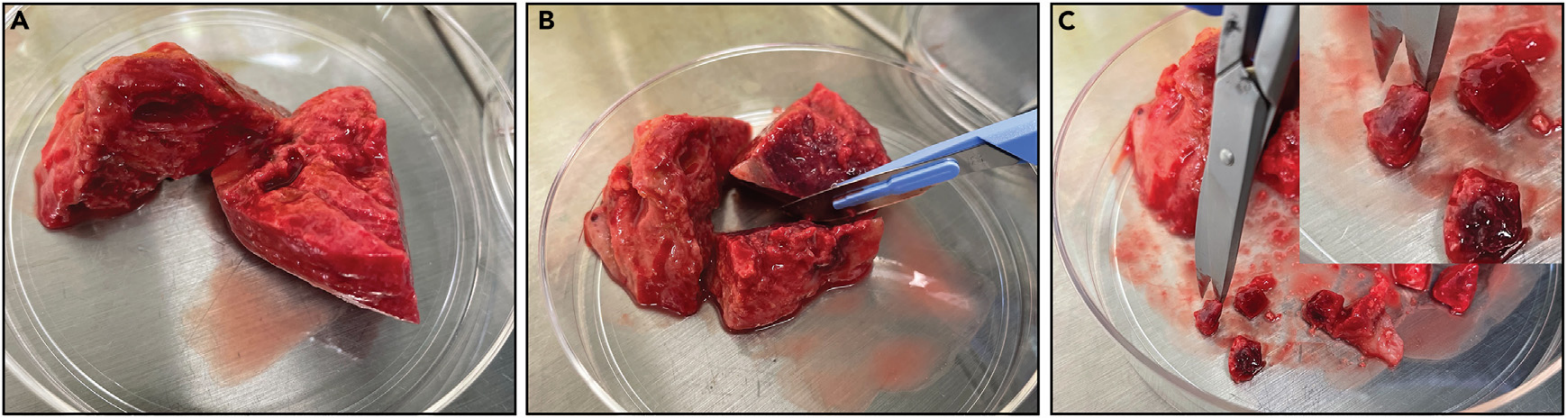
Sample handling for enzymatic tissue digestion Fresh tissue obtained from the surgery room is initially placed in a Petri dish (A). Subsequently, the tissue is cut into a 1 cm³ piece using a scalpel (B) and further fragmented into smaller portions using surgical scissors (C).
  f. Transfer the 12-well plate to the incubator, set at 37°C and 5% CO_2_, for at least 2 h before further use.
    **Pause point:** 3D Petri Dish micro-chips can be kept under sterile conditions in the incubator at 37°C and 5% CO_2_ or at 4°C for up to 7 days after the step 1e. Ovarian TumorMACS medium should be replaced every 2–3 days so that the 3D Petri Dish micro-chips do not dry out.

## Key resources table

**Table:** 

REAGENT or RESOURCE	SOURCE	IDENTIFIER
**Antibodies**		

E-Cadherin (4A2)/mouse (immunofluorescence 1:400)	Cell Signaling Technologies	Cat# 14472S; RRID:AB_2728770
Cleaved caspase-3 (D175)/rabbit (immunofluorescence 1:400)	Cell Signaling Technologies	Cat# 9661S; RRID:AB_2341188
Goat anti-mouse IgG (H + L) cross-adsorbed secondary antibody, Alexa Fluor 488 (immunofluorescence 1:500)	Invitrogen	Cat# A-11001; RRID:AB_2534069
Anti-rabbit IgG (H + L), F(ab')2 fragment (Alexa Fluor 647 conjugate) (immunofluorescence 1:500)	Cell Signaling Technologies	Cat# 4414S; RRID:AB_10693544
APC/Cyanine7 mouse IgG1, κ isotype ctrl antibody (flow cytometry 1:200)	BioLegend	Cat#400128; RRID:AB_2892538
REA control antibody (S), PE human IgG1, REAfinity (flow cytometry 1:200)	Miltenyi Biotec	Cat # 130-113-438; RRID:AB_2733893
CD326 (EpCAM) antibody, anti-human, PE, REAfinity (flow cytometry 1:200)	Miltenyi Biotec	Cat# 130-110-999; RRID:AB_2657495
APC/Cyanine7 anti-human CD45 antibody (flow cytometry 1:200)	BioLegend	Cat#304014; RRID:AB_314402
DAPI (4′,6-diamidino-2-phenylindole, dilactate) (flow cytometry 1:5,000)	BioLegend	Cat# 422801

**Biological samples**		

Ovarian cancer tissue and ascites	University Hospital Basel	N/A

**Chemicals, peptides, and recombinant proteins**		

Carboplatin 10 mg/mL = 27 mM	Labatec Pharma SA	Cat# 7680629020011
Paclitaxel 2 mM	Merck AG	Cat# T1912
PRT4165 (olaparib) 20 mM	Sigma-Aldrich	Cat# SML1013
Agarose, LE, analytical grade	Promega	Cat# V3125
Citrate buffer	Sigma-Aldrich	Cat# C9999
Accutase	Millipore	Cat# SRC005
Collagenase/dispase	Sigma-Aldrich	Cat# 10269638001
DNase I	NEB	Cat# M03033S
Red blood cell lysis solution 10×	Miltenyi Biotec	Cat# 170-080-033
Debris removal solution	Miltenyi Biotec	Cat# 130-109-398
Ovarian tumor MACS media	Miltenyi Biotec	Cat# 130-119-483
Ovarian tumor MACS media supplement	Miltenyi Biotec	Cat#130-119-483
Antibiotic antimycotic solution 100×, stabilized	Sigma-Aldrich	Cat# A5955
Formalin 4%	Formafix AG	N/A
Triton X-100	Sigma-Aldrich	Cat# T-8787
Fetal bovine serum (FBS)	Sigma-Aldrich	Cat# F7524
Tween 20	Sigma-Aldrich	Cat# P1379
ProLong gold antifade reagent with DAPI	Cell Signaling Technology	Cat# 8961
Ethanol absolute	Biosystems Switzerland	Cat# 179-VL54K
Ethanol 70%	Bichsel	Cat# 160006600
Xylol	Carl Roth	Cat# 9713.3
Dulbecco’s phosphate-buffered saline	Sigma-Aldrich	Cat# D8537
Citrate buffer 10×	Sigma-Aldrich	Cat# C9999
Albumin fraction V	Carl Roth	Cat# W8076.4
DMSO	Carl Roth	Cat# 4720
Trypan blue solution	Sigma-Aldrich	Cat# T8154
MACS tissue storage solution	Miltenyi Biotec	Cat# 130-100-008
Cover slides (24 × 50 mm, H&E)	Knitter Glass	Cat#VM52450Y1A.01
ClearVue mountant XYL (H&E)	Thermo Fisher Scientific	Cat#4212
Scott’s tap water working solution (H&E)	Leica Biosystems	Cat#3802901E
0.5% acid alcohol (H&E)	Leica Biosystems	Cat# 3803650E
UltraClear (xylene substitute, H&E)	Leica Biosystems	Cat#3905.9010PE
Harris’s hematoxylin (H&E)	Leica Biosystems	Cat# 3873.1000
Eosin 1%, aqueous solution (H&E)	Leica Biosystems	Cat# 84-0012-00
Aqua Millipore	N/A	N/A

**Software and algorithms**		

Image J (2.3.0/1.53q)	National Institutes of Health	Version 1.54f
QuPath	https://qupath.github.io/	Version 0.4.3
FlowJo	BD Biosciences	Version 10.10
RStudio	Posit, PBC	Version 3.6.1

**Other**		

Screw cap tube, 50 mL	Sarstedt	Cat# 62.547.255
Screw cap tube, 15 mL	Sarstedt	Cat# 62.554.502
1,250 μL biosphere filter tips	Sarstedt	Cat# 70.1186.210
200 μL filter tips	Sarstedt	Cat# 70.3031.255
20 μL filter tips	Sarstedt	Cat# 70.3030.265
Scalpel	Swann-Morton	Cat# 0506
Surgical scissors standard	HUBERLAB	3.7750.02
70 μm cell strainer	Falcon	Cat# 352350
Counting chambers, BLAUBRAND, improved Neubauer, bright-line, without clips, double ruling	Brand GmbH	Cat# 717810
MicroTissues 3D Petri Dish micro-mold spheroids	Sigma-Aldrich	Cat# Z764000-6EA
12-well clear flat bottom TC-treated multiwell cell culture plate	Falcon	Cat# 353043
Dead cell removal kit	Miltenyi Biotec	Cat# 30-090-101
15 mL reaction tubes	Miltenyi Biotec	Cat# 130-042-401
QuadroMACS separator	Miltenyi Biotec	Cat# 130-090-976
Minisart non-pyrogenic cellulose acetate filter 0.2 μm	Sartorius	Cat# 16534
Deparaffinization container	Bio-Optica Milano Spa	Cat# W05039099
HistoGel	Epredia	Cat# HG-400-012
ImmEdge hydrophobic barrier PAP pen (Dako pen)	Vector laboratories	Cat# H-4000
Epredia Superfrost Plus adhesion control microscope slide (75 × 25 × 1 mm)	Thermo Fisher Scientific	Cat# 22-042-941
Cover slips (24 × 60 mm)	Leica Biosystems	Cat# 85–0191-00
Plastic cassettes	Leica Biosystems	Cat# 81-0051-00
Rotary microtome (Microm HM 340)	Thermo Fisher Scientific	Cat# 387830
Blades for microtome S-35	Feather	Cat# 02.075.00.000
Spinning-disk CSU W1 confocal microscope	Nikon	N/A
New Brunswick Innova 42/42R	Eppendorf	N/A
Cytoflex S	Beckman Coulter Life Sciences	N/A
UltraRocker rocking platform	Bio-Rad	N/A
Gemini autostainer	Thermo Fisher Scientific	N/A
TPC 15 Duo/Trio	MEDITE Medical GmbH, Germany	N/A

## Materials and equipment

**Table:** Ovarian TumorMACS Medium, human

Reagent	Final concentration	Amount
Ovarian TumorMACS medium	N/A	48.5 mL
Ovarian TumorMACS medium supplement	2%	1 mL
Antibiotic Antimycotic Solution 100×	1%	500 μL
**Total**	**N/A**	**50 mL**

Store at 4°C for max. 2 weeks.

**Table:** Enzymatic tumor digestion mixture

Reagent	Final concentration	Amount
Ovarian TumorMACS growth medium	N/A	5 mL
Accutase	N/A	5 mL
Collagenase/Dispase	100 mg/mL	200 μL
DNase I	2 kU/mL	3 μL
**Total**	**N/A**	**10 mL**

Prepare freshly every time before use.

**Table:** Immunofluorescence staining: Blocking buffer

Reagent	Final concentration	Amount
BSA	1%	0.5 g
FBS	5%	2.5 mL
Triton X-100	0.1%	50 μL
PBS	N/A	47.45 mL
**Total**	**N/A**	**50 mL**

Filter with 0.2 μm filters, store at 4°C for max. 4 weeks.

**Table:** Immunofluorescence staining: Antibody solution

Reagent	Final concentration	Amount
BSA	1%	0.5 g
Triton X-100	0.1%	50 μL
PBS	N/A	49.95 mL
**Total**	**N/A**	**50 mL**

Filter with 0.2 μm filters, store at 4°C for max. 4 weeks.

### Short recipes

• 3% (w/v) agarose: Add 1.5 g of agarose, LE analytical grade to 50 mL of aqua millipore and autoclave.

Store at 4°C for max. 4 weeks.

• Flow cytometry buffer: Add 5 mL of FBS to 495 mL of PBS.

Store at 4°C for max. 4 weeks.

• Carboplatin 200 μM drug treatment solution: Add 74.1 μL of 27 mM carboplatin stock solution to 10 mL of Ovarian TumorMACS growth medium.

Use the same day.

• Carboplatin 100 μM drug treatment solution: Take 5 mL of carboplatin 200 μM drug treatment solution and add 5 mL of Ovarian TumorMACS growth medium.

Use the same day.

• Carboplatin 20 μM drug treatment solution: Take 400 μL of carboplatin 200 μM drug treatment solution and add 3600 μL of Ovarian TumorMACS growth medium.

Use the same day.

• Carboplatin 10 μM drug treatment solution: Take 700 μL of carboplatin 100 μM drug treatment solution and add 6300 μL of Ovarian TumorMACS growth medium.

Use the same day.

• Paclitaxel 1 μM drug treatment solution: Add 1 μL of paclitaxel stock solution at 2000 μM concentration to 2 mL of Ovarian TumorMACS growth medium.

Use the same day.

• Paclitaxel 0.2 μM drug treatment solution: Take 2000 μL of paclitaxel 1 μM drug treatment solution and add 8000 μL of Ovarian TumorMACS growth medium.

Use the same day.

• Paclitaxel 0.02 μM drug treatment solution: Take 400 μL of paclitaxel 0.2 μM drug treatment solution and add 3600 μL of Ovarian TumorMACS growth medium.

Use the same day.

• Paclitaxel 0.1 μM drug treatment solution: Take 5 mL of paclitaxel 0.2 μM drug treatment solution and add 5000 μL of Ovarian TumorMACS growth medium.

Use the same day.

• Paclitaxel 0.01 μM drug treatment solution: Take 700 μL of paclitaxel 0.1 μM drug treatment solution and add 6300 μL of Ovarian TumorMACS growth medium.

Use the same day.

• Combination – carboplatin 100 μM + paclitaxel 0.1 μM drug treatment solution: Take the same volume of carboplatin 200 μM drug treatment solution and paclitaxel 0.2 μM drug treatment solution and mix.

Use the same day.

• Combination – carboplatin 10 μM + paclitaxel 0.01 μM drug treatment solution: Take the same volume of carboplatin 20 μM drug treatment solution and paclitaxel 0.02 μM drug treatment solution and mix.

Use the same day.

• Olaparib 100 μM drug treatment solution: Add 8 μL of 100 mM olaparib stock solution to 8 mL of Ovarian tumor MACS growth medium.

Use the same day.

• Olaparib 10 μM drug treatment solution: Take 700 μL of olaparib 100 μM drug treatment solution and add 6300 μL of Ovarian tumor MACS growth medium.

Use the same day.

• Immunofluorescence staining – Permeabilization buffer: Add 125 μL of Triton X-100 to 50 mL of PBS.

Filter with 0.2 μm filters, store at 4°C for max. 4 weeks.

• Immunofluorescence staining – Wash buffer: PBS-T: Add 500 μL of Tween 20–500 mL of PBS.

Filter with 0.2 μm filters, store at 4°C for max. 4 weeks.

## Step-by-step method details

### Handling, processing, and seeding of fresh patient-derived ascites and tissue samples (optional: slow frozen samples)

**Timing: 4 h followed by 24 h incubation**

This section outlines the preparation and seeding of cell suspensions from patient-derived ascites and tissue samples into 3D Petri Dish micro-chips. The goal is to prepare a cell suspension suitable for downstream drug treatment experiments.

1. Isolation of patient-derived cells from ascites samples.
  a. Centrifuge freshly collected ascites at 400 × *g* for 15 min, remove supernatant.
  b. Resuspend cells pellet in 3 mL of pre-warmed at 37°C Ovarian TumorMACS growth medium.
  c. Check for the content of red blood cells, cell debris, and dead cells by visually inspecting the cell pellet and using a bright-field microscope.
  d. Count the cells:
    i. Mix 20 μL of the cell suspension with 20 μL of trypan blue solution.
    ii. Add 10 μL of the mix to the Neubauer counting chamber.

***Optional:*** If you use slow frozen ascites, resuspend the frozen ascites cells with 1 mL of pre- warmed at 37°C Ovarian TumorMACS growth medium in the cryotube. Transfer the cell suspension to a 15 mL tube and add 1 mL of Ovarian TumorMACS growth medium to obtain a total volume of 3 mL. Continue with step 1c.

2. Isolation of cells from tissue samples (e.g., omentum, minimum amount of 3 g of tissue).
  Freshly collected tissue should be processed within 30 min after collection. For longer periods, the tissue should be stored in MACS Tissue Storage Solution for up to 48 h. Before continuing with the step 2a of the protocol, remove the MACS Tissue Storage Solution and wash the tissue twice with PBS.
  ***Optional:*** If you use slow frozen tissue samples, prepare two 50 mL tubes with 10 mL of Ovarian TumorMACS growth medium. Thaw the tissue in the cryotube for 1 min at 37°C inside a water bath. Add 1 mL of Ovarian TumorMACS growth medium and transfer the tissue to the first, then to the second tube containing Ovarian TumorMACS growth medium (to remove DMSO, present on the freezing media). Continue the protocol from step 2a.
  a. Prepare a 50 mL tube with 10 mL of the enzymatic tumor digestion mixture.
  b. Cut the tumor tissue (Figure 2A) first with a scalpel (Figure 2B) and then with surgical scissors (Figure 2C) in a petri dish until obtain pieces smaller than 8 mm^3^. Transfer the tumor pieces with a 1000 μL pipette into the 50 mL tube containing the enzymatic tumor digestion mix.
  c. Shake the tube for 1 h at 37°C using an orbital shaker.
  d. After incubation, add 10 mL of PBS to the tube containing the digested tissue.
    ***Note:*** Steps 2d–2j are performed at 20°C–22°C.
  e. Centrifuge at 300 × *g* for 10 min, then remove the supernatant using a 5 mL pipette.
    ***Note:*** Fat tissue can float on the surface and should be discarded.
  f. Resuspend the cell pellet in 20 mL of PBS.
  g. Filter the suspension through a 70 μm cell strainer into a new 50 mL tube.
  h. Centrifuge at 300 × *g* for 10 min, then remove the supernatant.
  i. Resuspend the cells in 3 mL of pre-warmed Ovarian TumorMACS growth medium at 37°C.
  j. Check for the content of red blood cells, cell debris, and dead cells.
  k. Count the cells:
    i. Mix 20 μL of the cell suspension with 20 μL of Trypan blue.
    ii. Add 10 μL of the mix to the Neubauer counting chamber.
      ***Optional:*** If you want to evaluate the exact immune and tumor cell content as well as the cell viability you can perform the following flow cytometry protocol. Take 50.000 cells in 120 μL of TumorMACS growth medium and add to each well of a V-shaped 96-well plate (minimum of 4 wells). Centrifuge for 4 min at 400 × *g* at 20°C–22°C. Remove the supernatant using the vacuum pump and re-suspend the cell pellet in 120 μL of flow cytometry buffer. Repeat the centrifugation and supernatant removal steps two times. Incubate the cells with the following conditions: A1 and A2: only flow cytometry buffer (control gating), A3: APC/Cyanine7 Mouse IgG1, κ Isotype Ctrl Antibody; REA Control Antibody, human IgG1, REAfinity -PE diluted 1:200 in flow cytometry buffer; A4: APC/CY7 Anti-human CD45 Clone H130, CD326 EpCAM-PE, Clone REA764 C, diluted 1:200 in flow cytometry buffer. Incubate for 30 min on ice, protected from the light. Then centrifuge for 4 min at 400 × *g* and remove the supernatant. Repeat this step twice. Resuspend the pellets from A2-A4 in flow cytometry buffer containing DAPI (diluted 1:5000)- DAPI 1 mg/mL. A1 must be resuspended in flow cytometry buffer only. Incubate for 5 min at 20°C–22°C. Centrifuge for 4 min at 400 × *g*. Re-suspend the cell pellets in flow cytometry buffer and run on the CytoFLEX S flow cytometer. An example of the flow cytometry analysis is provided in Figure 3.

**Figure 3:**
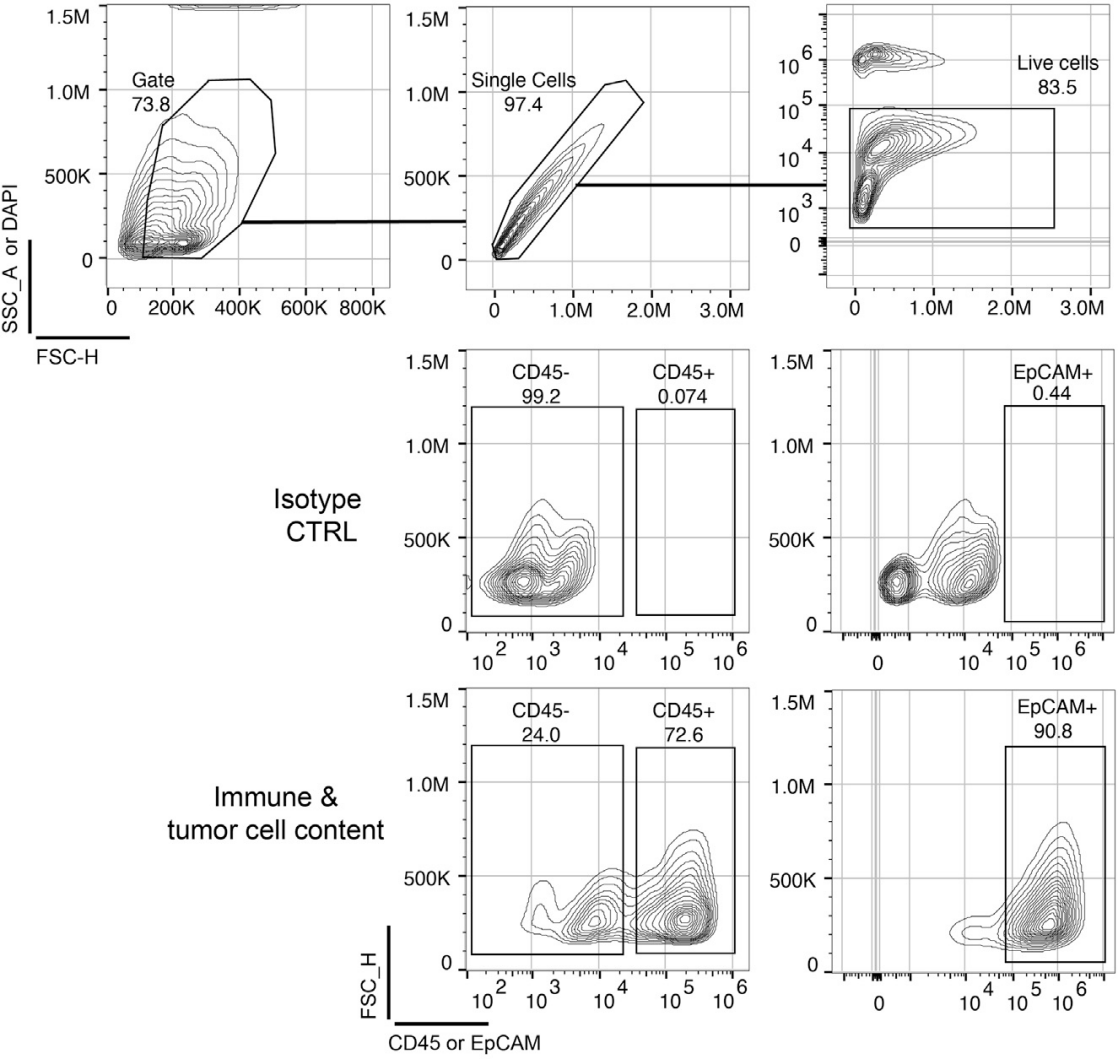
Flow cytometry evaluation of immune cell and tumor cell content and cell viability Flow cytometry plot showing the initial gating strategy based on forward scatter height (FSC_H) and side scatter area (SSC_A) to exclude debris and ensure single-cell events. The gated population is subsequently analyzed for immune and tumor cell content.
3. Preparation of patient-derived cell suspension.
  a. Prepare a cell suspension at a density of 1.25 × 10^6^ cells/mL, 2.5 × 10^5^ cells in 200 μL per condition.
    Example: For 10 conditions: 10 × 2.5 × 105 = 2.5 million cells are needed.
  b. Spin the cell suspension at 300 × *g* for 5 min.
  c. Resuspend the cell pellet in 200 μL of Ovarian TumorMACS growth medium per condition. Example: Resuspend in 10 × 200 μL = 2000 μL Ovarian TumorMACS growth medium.
    ***Note:*** If the final number of cells exceeds the number required for the planned experiments, isolated cells can be frozen in freezing medium (90% FBS and 10% DMSO) in cryovials containing 0.5–3 million cells per cryovial and stored at −80°C until further use.
4. Seeding of patient-derived cells in 3D Petri Dish micro-chips.
  a. Take the 12-well plate with the prepared 3D Petri Dish micro-chips out of the incubator.
  b. Remove culture medium surrounding the chips.
  c. Use the 200 μL pipette and carefully remove the medium inside the 3D Petri Dish micro-chips.
    ***Note:*** Avoid touching the surface of the chip.
  d. Fill each chip with 200 μL of the cell suspension.
  e. Let the cells slowly settle down for 20 min.
  f. Check under the microscope if the cells settled at the bottom of the wells of the 3D Petri Dish micro-chips (Figures 4A and 4B).
    ***Note:*** If the 3D Petri Dish micro-chip is damaged and the cells are not in the well (Figures 4C–4E) you need to discard the micro-chip and start over.

**Figure 4:**
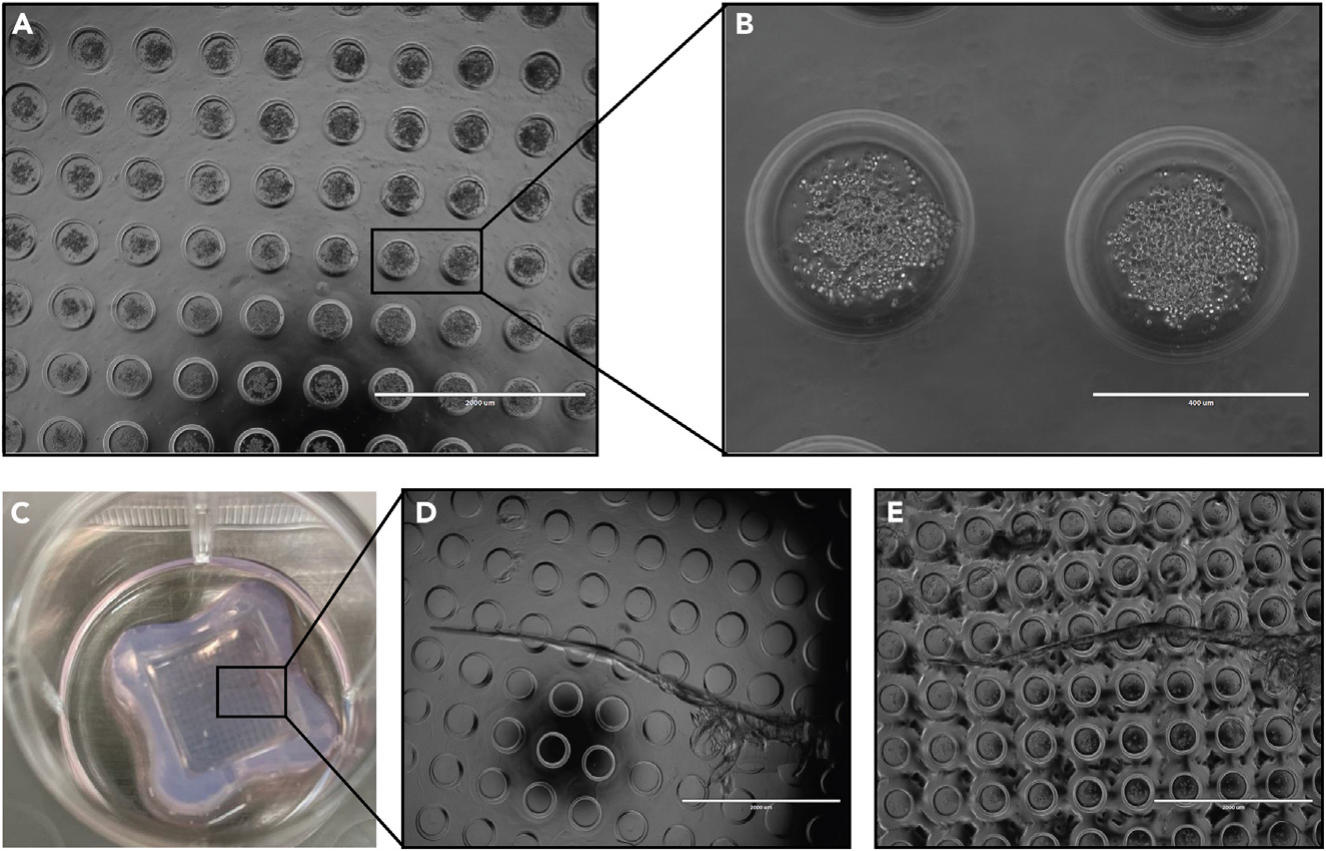
3D Petri Dish micro-chips containing patient-derived cells The cells are adequately distributed at the bottom of the micro-chip wells (A and B). An image of a damaged micro-chip with a leak, depicting cells that are outside of the micro-chip wells, which needs to be discarded (C–E).
  g. Add 2 mL of Ovarian TumorMACS growth medium to the well of the 12-well plate.
  h. Put the 12-well plate back into the incubator at 37°C and 5% CO_2_.
  i. Incubate for 24 h before continuing with major step two.

### Drug treatment

**Timing: 3 h followed by 48 h incubation**

This section covers the procedures for administering drug treatments to the patient-derived cells seeded in 3D Petri Dish micro-chips after an initial incubation period.

5. Treatment of the cells after 24 h of incubation time.
  a. Take the 12-well plate out of the incubator and check the 3D Petri Dish micro-chips under the microscope.
  b. Carefully remove the Ovarian TumorMACS growth medium surrounding the chip avoiding to disturb the cells.
  c. Add 2 mL of the designated drug and concentration to each well.
  d. Put the 12-well plate back into the incubator for 48 h at 37°C and 5% CO_2_.

### Fixation, paraffin embedding, and sectioning

**Timing: 3 days**

The step begins with formalin fixation, which stabilizes cellular structures in the 3D Petri Dish micro-chips. Subsequent steps include tissue processing, paraffin embedding, and microtomy to produce uniform tissue sections for downstream immunofluorescence staining.

6. Formalin fixation.
  a. After 48 h of treatment, remove the medium surrounding the 3D Petri Dish micro-chips in the 12-well plate.
  b. Wash every well with 2 mL of PBS.
  c. Add 2 mL of 4% formalin to each well and incubate for 20 min in the cell culture hood at 20°C–22°C.
  d. Wash two times with 2 mL of PBS per well.
  e. Remove the PBS surrounding the chip, as well as on top of the chip (∼ 200 μL).
    ***Note:*** The removal of the liquid on the top of the chip needs to be performed carefully since the chips should not be damaged.
    ***Note:*** Steps a-e should be done in the cell culture hood and afterwards, sterile conditions are no longer needed.
  f. Carefully, cover each chip with 200 μL of histogel (Figures 5A and 5B).
    ***Note:*** Histogel should be previously liquified using the microwave at 450 W for 20–30 s. Loosen the lid of the histogel tube slightly, and exercise extra caution when heating the histogel since it can boil over. Therefore, it is recommended to repeat the heating process at least once for a short period of time (5–10 s) to ensure liquification of the histogel. Avoid the formation of air bubbles to have a uniform layer of histogel on top of the cells.

**Figure 5:**
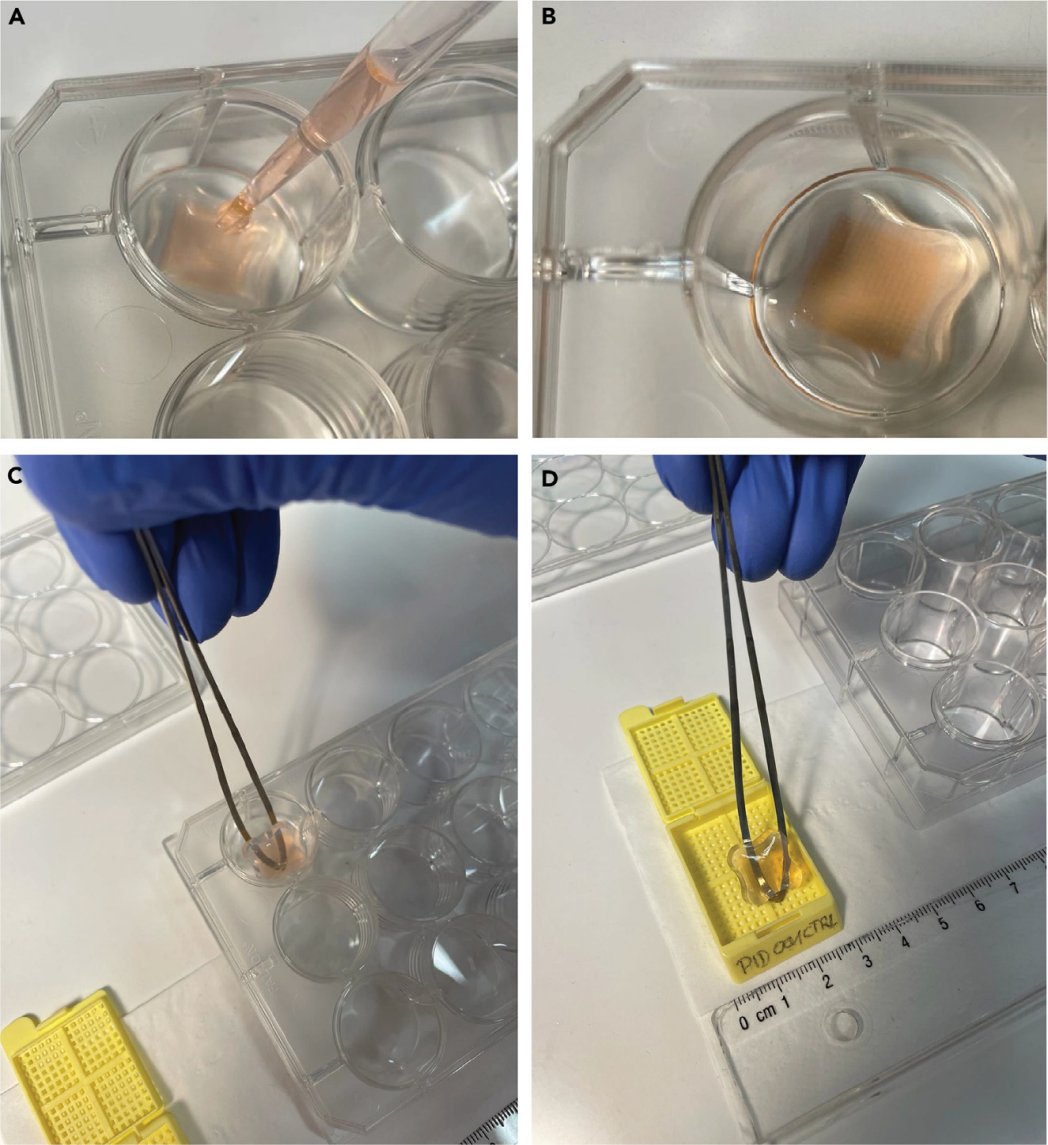
3D Petri Dish micro-chips handling for paraffin embedding Application of Histogel (A and B). Transfer of the 3D Petri Dish micro-chip to a pre-labeled histological cassette (C and D).
  g. Let the histogel harden for 5–10 min at 20°C–22°C.
  h. Transfer each 3D Petri Dish micro-chip to a separate pre-labeled histological cassette, with the help of a sterile spatula (Figures 5C and 5D).
    ***Note:*** Histological cassettes need to be labeled with a pencil due to the following procedure that involves different ethanol concentrations.
  i. Move the cassettes to a container filled with 70% ethanol.
    **Pause point:** Cassettes can also be stored in ethanol 70% for up to 4 days at 4°C.
7. Tissue processing with tissue processor TPC 15 Duo/Trio (overnight).
  The tissue processor has a predefined protocol that runs in an automated manner as follows.
  a. 70% ethanol for 45 min.
  b. 80% ethanol for 45 min.
  c. 96% ethanol for 1 h and 15 min.
  d. 100% ethanol for 30 min.
  e. 100% ethanol for 1 h.
  f. 100% ethanol for 1 h.
  g. Xylene for 30 min.
  h. Xylene for 45 min.
  i. Xylene for 1 h.
  j. Paraffin 62°C for 1 h.
  k. Paraffin 62°C for 1 h and 45 min.
  l. After the last cycle is finished, transfer the cassettes onto the warm plate of the paraffin embedding machine.
8. Paraffin embedding.
  a. Take a histological cassette from the warm plate and remove the cassette lid in order to view the cells embedded in the 3D Petri Dish micro-chip.
  b. Select a mold that best corresponds to the size of the 3D Petri Dish micro-chip.
    ***Note:*** The 3D Petri Dish micro-chip should not touch the edge of the mold.
  c. Transfer the histological cassette containing the chip onto the warm plate.
  d. Pour melted paraffin from the paraffin dispenser until the mold is partially filled.
  e. With a forceps, remove the 3D Petri Dish micro-chip from the cassette and place it at the bottom of the mold.
  f. Embed the 3D Petri Dish micro-chip flat against the mold surface with enough pressure to obtain a flat homogeneous surface for getting a complete section later on.
    ***Note:*** All sides of the 3D Petri Dish micro-chip must be surrounded by at least 2 mm of paraffin for maximum cutting support.
  g. Carefully transfer the mold onto the cold plate. Allow the paraffin to solidify for a few seconds until it turns white.
  h. Immediately place the original labeled embedding cassette on top of the embedding mold. Note: Use the original embedding cassette, which is labeled with the patient identification number and condition.
  i. Carefully fill the combined mold and embedding cassette with paraffin to the level of the upper edge of the cassette.
  j. Cool immediately by transferring the mold and cassette onto the cold plate.
  k. Wait at least 3–4 h so the paraffin is hardened completely before you separate the mold from the embedding cassette.
  l. The paraffin block is now ready for sectioning.
    **Pause point:** Paraffin blocks can be stored for several years at 20°C–22°C.
9. Microtomy: Sectioning formalin-fixed, paraffin-embedded samples.
  a. Remove the excess of paraffin from the sides of the histological cassettes by scraping or melting.
  b. Place the paraffin blocks onto the cold plate before trimming.
  c. Carefully insert the blade into its holder on the microtome, ensuring that the blade is positioned correctly.
  d. Release the clamp of the paraffin block holder by pulling the vertical lever forward and snap the cassette into the cutting position.
    ***Note:*** Always make sure that the hand wheel brake is on before inserting paraffin blocks into the holder.
  e. Release the brake (lever/button) on the hand wheel.
  f. Using the coarse advance wheel, bring the block close, up to the blade.
  g. When the block is close to the blade, use the trimming button to advance, and cut into the paraffin block until a full face of the 3D Petri Dish micro-chip is exposed. Trimming is done at a thickness of 10 μm to avoid the unnecessary loss of the material.
    ***Note:*** The last few trimming sections should be cut at the designated final thickness of 5 μm to polish the block surface.
  h. Once trimmed, place the paraffin block face down on the ice block and leave it there until it is thoroughly chilled (3–5 min).
  i. Replace the trimming blade with a new blade.
  j. Once the block is chilled, cut sections at 5 μm thickness.
    ***Note:*** Ensure that the block is oriented in the same way as it was for the trimming process.
  k. Turn the hand wheel at a steady rhythm.
  l. Paraffin sections will now slide down into the water bath.
    ***Note:*** The water bath should be heated at 40°C to fully extend the sections.
  m. Use a clean paint brush to smooth out the paraffin sections.
  n. Use a glass slide prelabeled to match the patient number and condition to pick up the individual paraffin sections by immersing the slide vertically into the water bath up to three quarters of its length, and then bringing the tissue section in contact with the slide.
    ***Note:*** Discard sections with wrinkles or bubbles.
  o. Place the slide in a rack to drain.
  p. Dry the slides for 12 h at 42°C before starting the antigen retrieval and staining process.
    **Pause point:** The slides with the paraffin sections can be stored at 20°C–22°C for several months.

### H&E and immunofluorescence staining

**Timing: 2 days in total**

This step involves Hematoxylin and Eosin (H&E) staining to visualize cellular architecture and content. Immunofluorescence staining follows, utilizing specific antibodies to detect tumor cells and evaluate treatment effects like apoptosis. These techniques prepare samples for detailed confocal microscopy, enabling comprehensive analysis of cellular responses to treatments.

10. H&E staining with the Gemini auto Stainer according the predefined protocol:
  a. Three-times Xylene for 2 min each.
  b. 100% alcohol for 2 min.
  c. 96% alcohol for 2 min.
  d. 70% alcohol for 2 min.
  e. 60% alcohol for 2 min.
  f. Distilled water for 30 s.
  g. Hematoxylin for 2 min.
  h. Tap Water for 1 min.
  i. Tap Water two times for 45 s.
  j. HCl (acid alcohol) for 2 min.
  k. Three-times tap water for 30 s each.
  l. Scott’s Tap Water for 2 min.
  m. Two-time distilled water for 30 s each.
  n. Eosin (Eosin 200 mL and Erythrosine b 1% 100 mL) for 4 min.
  o. Three-times tap water for 1 min each.
  p. Distilled water for 2 min.
  q. Three-times 96% ethanol for 5 min each.
  r. 100% ethanol for 5 min.
  s. Xylene for 5 min.
  t. Xylene for 2 min.
  u. Slides were mounted using ClearVue mountant XYL and a 24 mm × 50 mm glass coverslip.
11. Deparaffinization and rehydration for immunofluorescence staining according to Figure 6.

**Figure 6:**
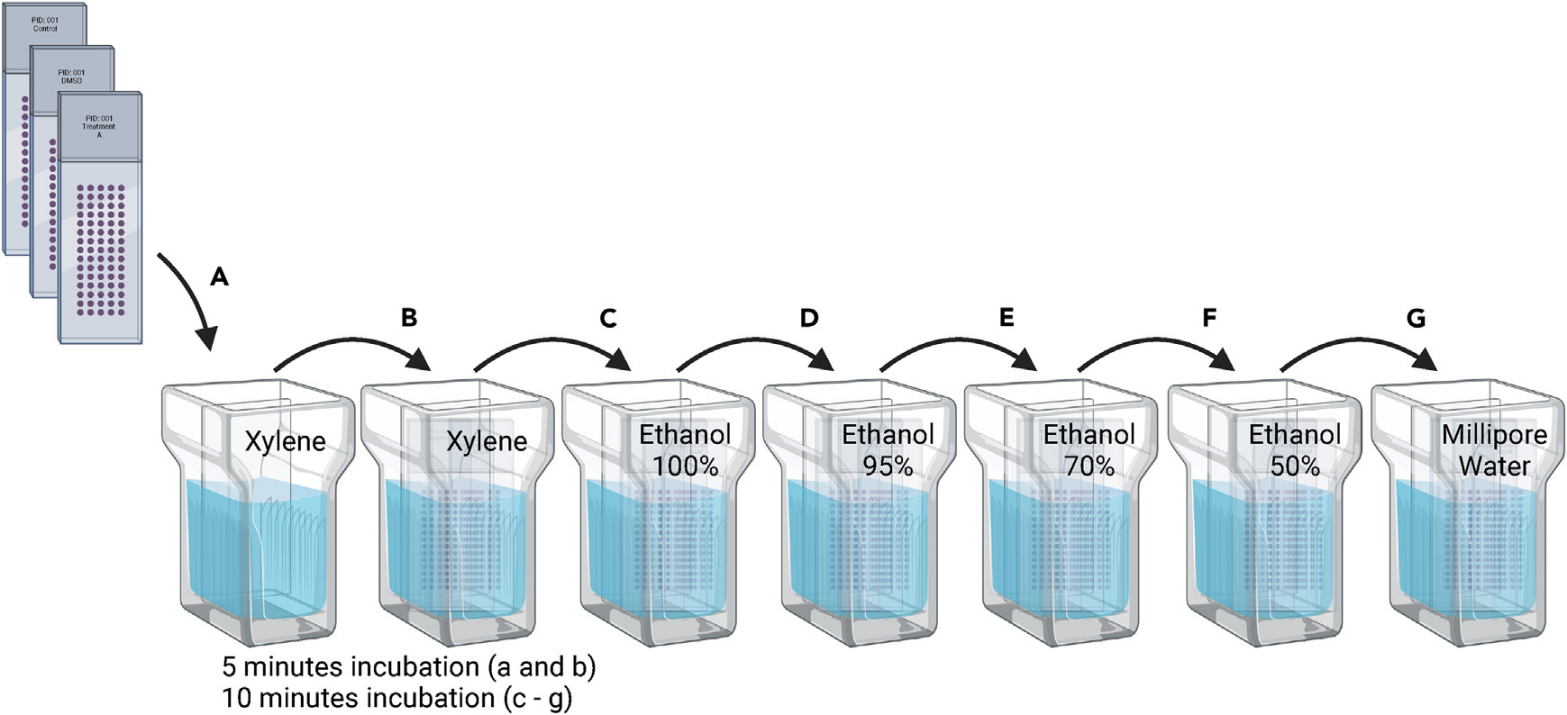
Deparaffinization and rehydration process for FFPE sections with embedded samples Place the slides containing cells into a slide holder. Transfer the slide holder to a container filled with xylene (A) for 5 min, followed by another container with xylene (B) for an additional 5 min. Subsequently, move the slide holder to a container containing 100% ethanol (C) for 10 min, then to 95% ethanol (D) for another 10 min, followed by 70% ethanol (E) for 10 min, and finally to a container with 50% ethanol (F) for 10 min. Transfer the slide holder to a container with Millipore water (G). This procedure should be conducted under a fume hood.
12. Antigen unmasking.
  a. Pre-boil 1× citrate buffer (300 mL) in the microwave for 5 min at 800 W.
  b. Place slides in the heat resistant container containing the pre-boil 1× citrate buffer and boil in the microwave for additional 5 min at 800 W.
    ***Note:*** To not boil over and therefore spill any citrate buffer, boil two times for 2.5 min, if necessary, readjust the volume of citrate buffer to always cover the glass slides.
  c. Add more citrate buffer to ensure that all the slides are covered.
  d. Boil for a second time for 5 min at 800 W.
    ***Note:*** See step 12b.
  e. After boiling for the second time, fill the heat resistant container up to the top with PBS, without removing the citrate buffer.
  f. Allow to cool down until the heat resistant container can be touched, for a maximum of 1 h at room temperature (20°C–22°C).
13. Antibody staining.
  a. Move slides from the heat resistant container to the slide holder containing PBS.
  b. Take slide by slide and remove the excess of PBS by gently tapping the edge of the slide onto tissue paper.
    ***Note:*** Avoid touching the area containing the sample.
  c. Use a hydrophobic pen to draw a circle around the area containing the samples.
    ***Note:*** Avoid touching the samples with the hydrophobic pen and apply generously to prevent liquid surpassing the borders.
  d. Permeabilize the cells by adding 200 μL of the permeabilization buffer and incubate for 5 min at 20°C–22°C. Avoid extended incubation time.
    ***Note:*** Liquids are always added to the center of the sample and all pipetting steps must be done carefully as the sample cells can detach from the slide.
  e. Move slides to a slide holder filled with PBS and wash for 2–5 min.
  f. Remove the PBS by gently tapping the edge of the slide onto tissue paper.
    ***Note:*** For removal of liquids, tilt the slide and remove the liquid carefully with the help of tissue paper.
  g. Add 250 μL of blocking buffer.
  h. Incubate for 1 h at 20°C–22°C.
  i. Remove the blocking buffer from the slides by gently tapping the edge of the slide onto tissue paper.
  j. Dilute primary antibody in the antibody solution according to the manufacturer’s instructions to obtain a final of 250 μL per slide.
  k. Incubate for 16 h at 4°C.
  l. The next day, wash with PBS-T for 10 min on the orbital shaker (Ultra Rocker, Rocking platform). Repeat once.
  m. Wash once with PBS for 10 min on the orbital shaker.
  n. Remove PBS from the slides by gently tapping the edge of the slide onto tissue paper.
  o. Dilute the secondary antibody in the antibody solution according to the manufacturer’s instructions to have a final volume of 250 μL per slide.
    **CRITICAL:** The secondary antibody solution is light sensitive, so make sure that the slides are protected from light.
  p. Incubate for 3 h at 20°C–22°C in a light-protected container.
  q. Wash the slide by immersion into PBS-T in a light-protected container for 20 min. Repeat this step once.
  r. Wash once with PBS only in a light-protected container for 20 min.
  s. Remove any excess liquid from the slide by gently tapping the edge of the slide onto tissue paper.
  t. Add 50 μL of ProLong Gold Mounting medium (containing DAPI) in the middle of the circle that you drew using the hydrophobic pen.
  u. Carefully cover the sample with a 24 mm × 36 mm × 0.17 mm coverslip.
    ***Note:*** Avoid trapping any air bubbles under the coverslip, as later the imaging will not be possible at this spot.
  v. Seal the edges of the coverslip with transparent nail polish and allow the mounting medium to cure for 5 min in the dark.
  w. Allow the slide to cure for 1 h at 4°C in the dark before continuing with the image acquisition.
    **Pause point:** The slides can be stored at 4°C in the dark for up to 4 weeks.

### Image acquisition and analysis

**Timing: 2 days in total**

This step involves acquiring high-resolution confocal images of cellular structures and fluorescence signals from the cell aggregates. These images are meticulously analyzed using Image J and QuPath software to quantify DAPI-positive cells, tumor cells, and apoptotic cells. The analysis is further supported by the DRUGSENS r-package developed for this protocol, enabling comprehensive assessment of treatment effects and providing valuable data for further analysis and interpretation.

#### Part 1

**Figure 7:**
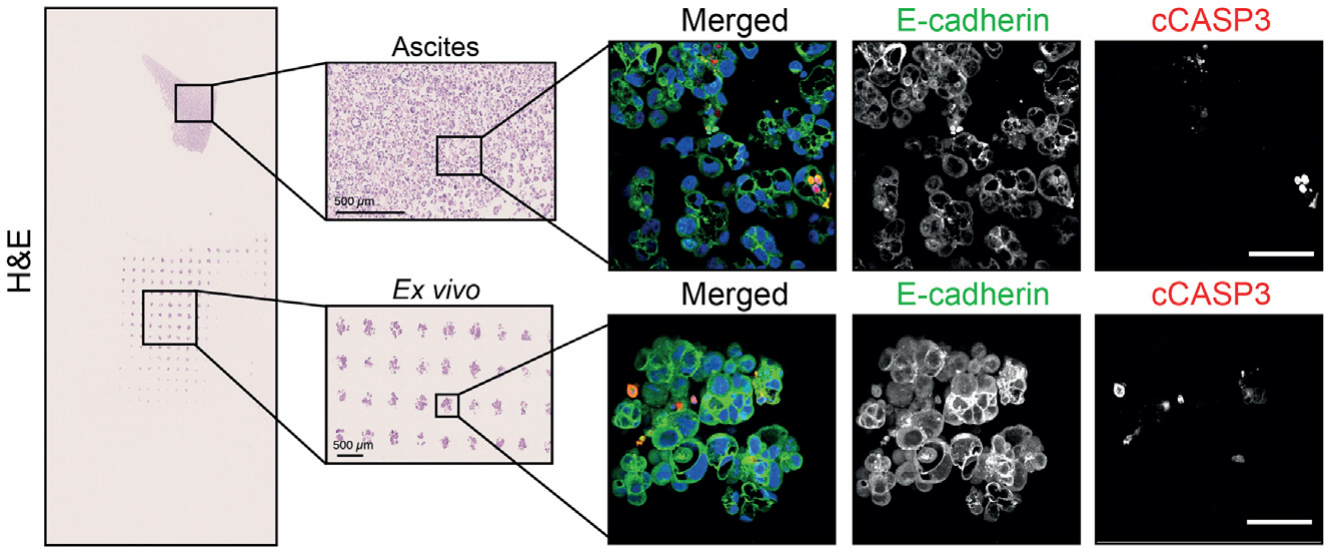
Representative hematoxylin and eosin (H&E) staining and immunofluorescence staining for tumor (E-Cadherin) and apoptotic (cleaved caspase-3 = cCASP3) cells from ascites (original) and *ex vivo* cultures Scale bars: 500 μm (H&E) and 50 μm (immunofluorescence).

Image acquisition (minimum 15 aggregates imaged, 15–20 min per slide) (Figure 7).

14. Place the slide on the confocal microscope (Nikon Ti microscope with Yokogawa CSU-W1 spinning disk unit 50 μm pinhole, and a Teledyne Photometrics Prime 95B (pixel cell size of 16 μm × 16 μm). To ensure best camera chip illumination, we captured images using the center 1200 × 1200 camera pixel (fitting the Nikon Ti field of view of 18 mm diagonal) stage and locate the area of interest under a low magnification.

***Note:*** Handle slides carefully to avoid smudging or damaging the sample.

Set appropriate imaging parameters for optimal image quality with a defined exposure time for each channel for all the images acquired allowing a comparable quantification of the signal intensity For the Nikon CSU-W1 confocal microscope we recommend to use Plan Apo λ 10× numerical aperture (NA) = 0.45 objective for sample localization, and a Plan Apo λ 40× NA = 0.95 objective for image acquisition.

***Note:*** It is vital to always apply identical acquisition settings including exposure time, number of z-slices and spacing, bit depth, magnification, resolution, and binning for each channel and for all the images acquired. The CSU-W1 was equipped with a dichroic mirror DM405/488/561/640, and following emission filters in the filter wheel: ET460/50 m, ET525/50 m, ET630/75 m and ET700/75 m. A laser light source LightHUBplus (Omicron-Laserage) with 405 nm, 488 nm, 561 nm and 640 nm lasers was used. DAPI was excited with a 405 nm laser and the emission light was filtered with an ET460/50 m emission filter. Alexa Fluor488 was excited with a 488 nm laser and the emission light was filtered with an ET525/50 m emission filter. Alexa Fluor 647 was excited with a 647 nm laser and the emission light was filtered with an ET700/75 m emission filter. The laser power was set at 100% and the histogram was optimized using the camera exposure time.

15. Make sure the microscope focuses correctly on every spheroid before starting the automated image acquisition process. We recommend using Nikon NIS AR for automated image acquisition.
16. Acquire 11 slices per image stack (5 μm up and down from the plane of interest, in 1 μm steps) to have multiple focal planes.
17. Acquire at least 15 micro-wells/ spheroids per condition.

#### Part 2

Image analysis (3–4 h per slide).

**Figure 8:**
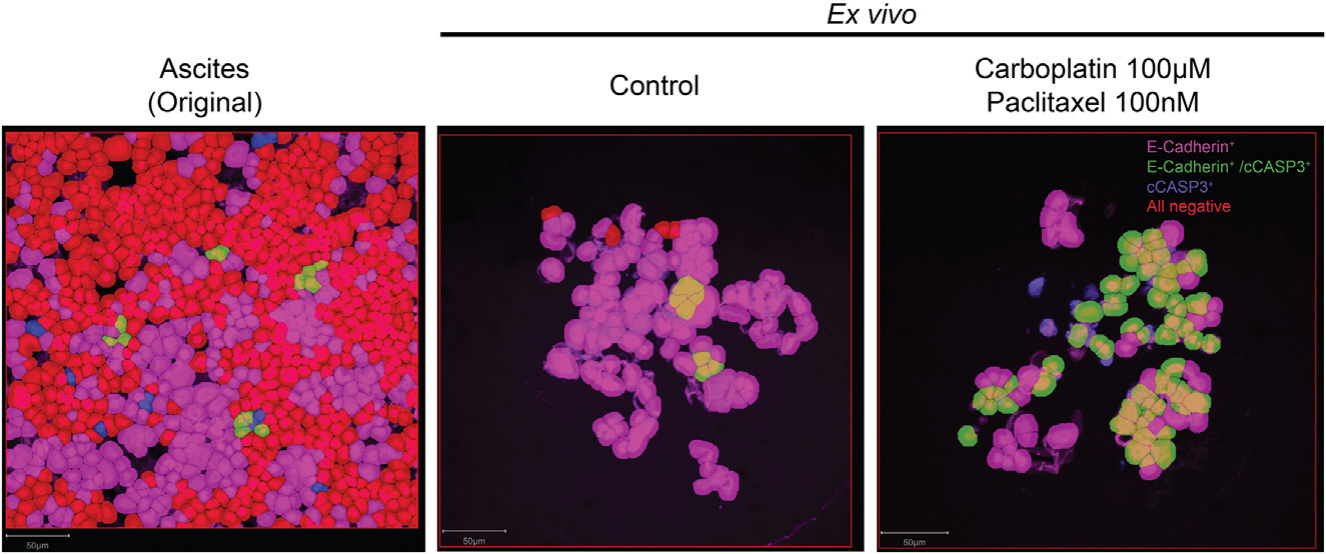
QuPath classification of different cell types DAPI only positive cells are displayed in red, E-Cadherin only positive cells are displayed in magenta and double-positive cells (E-Cadherin+ and cCASP3+) are displayed in green. Scale bar: 50 μm.

**Figure 9:**
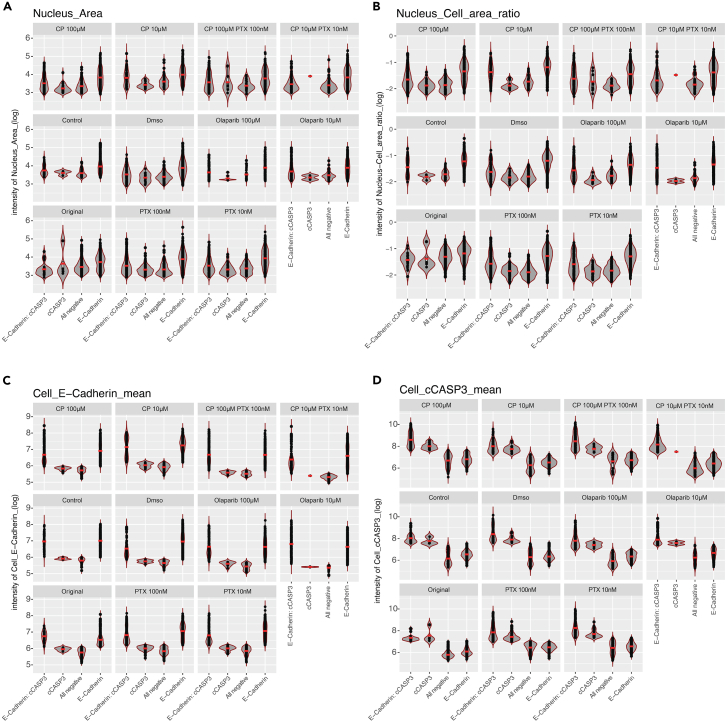
Quality control plots Different QuPath classifiers analyzing the intensity of the nucleus area (A), the nucleus cell area ratio (B), E-Cadherin (C), and cCASP3 (D).

**Figure 10:**
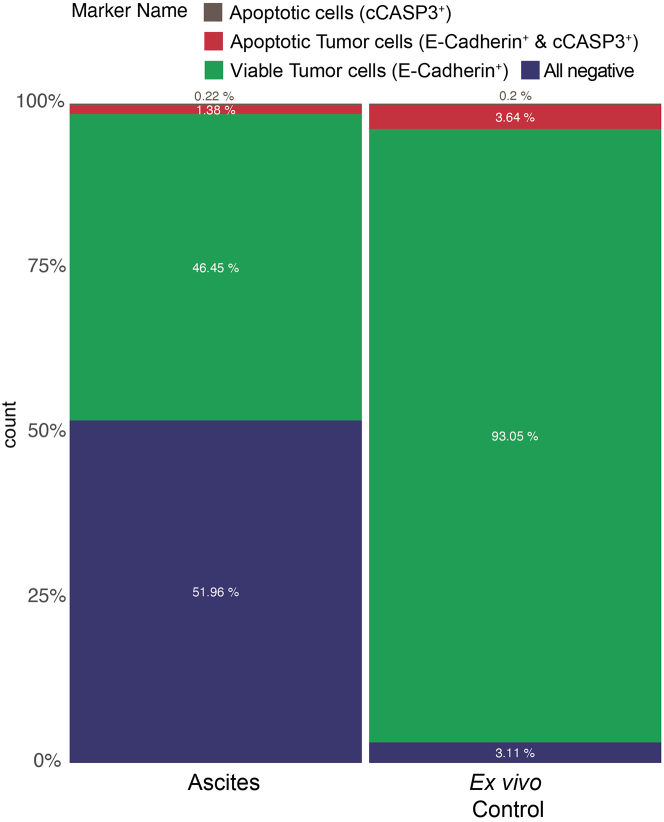
Cell composition of the original sample (ascites) and *ex vivo* (control) in percentage On the left is the cell composition of the original sample (ascites) depicted with a balanced distribution of other cells, all negative (blue) and tumor cells E-Cadherin positive (green) and only very few double-positive cells (red) and apoptotic cells (brown). Due to the tumor cell enhancing media the ratio between all negative and E-Cadherin positive cells shifts in the *ex vivo* model.

**Figure 11:**
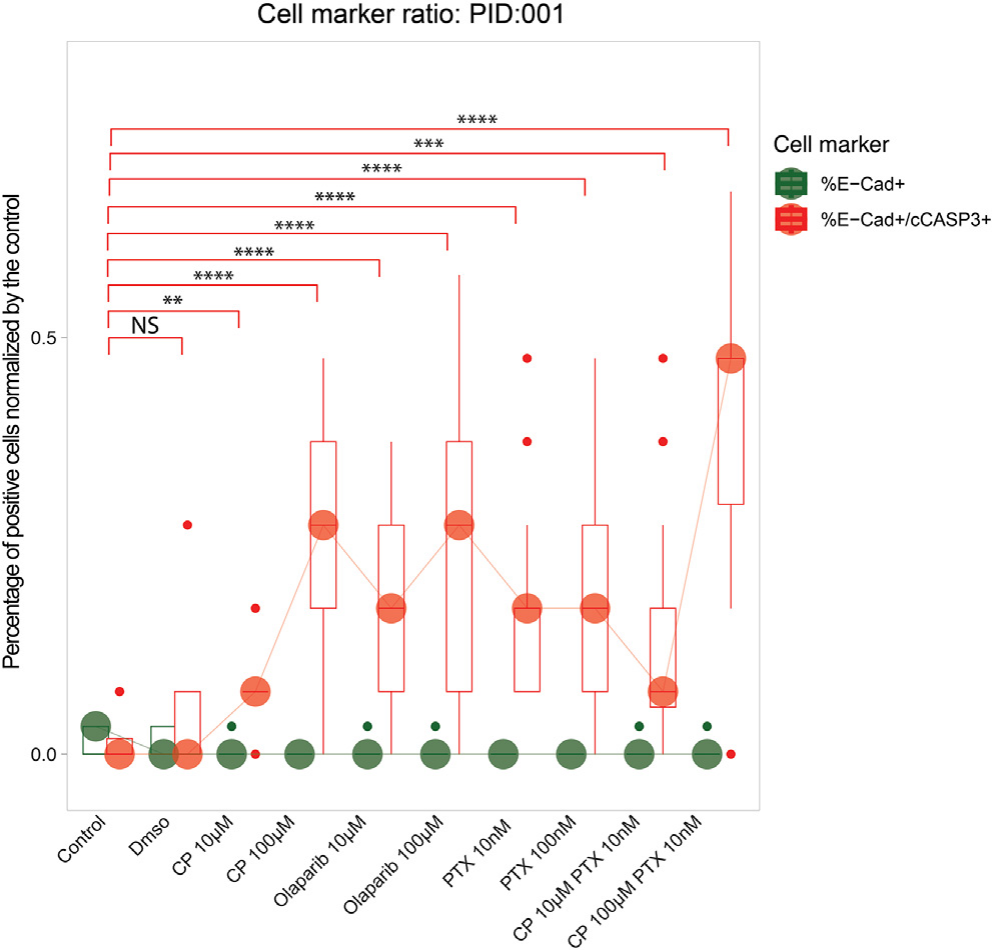
Drug efficacy normalized to control in an *ex vivo* model Example for drug efficacy of two different concentrations of carboplatin (CP), olaparib, paclitaxel (PTX), and the combination of carboplatin and paclitaxel (CP PTX) in an ex *vivo* sample normalized to control. ∗∗*p* < 0.01, ∗∗∗*p* < 0.001, ∗∗∗∗*p* < 0.00001.
  d. Run the code to identify the correct cells in every single image.

***Note:*** You might need to adjust your settings and raise / lower your detection threshold.

18. Merge the individual z-stack images to a z-stack projection, using the average intensity signal in each z-slice, into one .tiff-file using Image J software.^7^
19. Import stacked images to QuPath^8^ by creating a new “project” in a designated folder.
20. Choose “fluorescence” as your set image type.
21. Use the “rectangle” annotation tool to outline and annotate your region of interest within the image.
22. Import the following scripts from your desktop: “Image_workflow_.groovy” and “ExportMeasurements”. Scripts and further description are available through the DRUGSENS r package.
23. Use the script “Image_workflow_.groovy” that identifies DAPI-positive cells (cell identification).
  a. Utilize QuPath’s cell detection tools to identify and segment individual cells within the annotated region.
  b. Adjust cell detection settings (cell size, detection sensitivity) as needed for optimal results.
  c. Manually create a threshold for positive / negative image detection of tumor cells and apoptotic cells by creating two single measurement classifiers (Figure 8). Additional details are provided through the DRUGSENS r package. Exemplified quality control plots for the staining intensity of the different markers across various treatments, cell type quantification, and the effects of the different treatments with regard to tumor cell apoptosis are presented in Figures 9, 10, and 11, respectively.

24. Once all the cells are correctly detected, use the script “ExportMeasurements” to export your data as a CSV file for downstream analysis.

## Expected outcomes

This protocol is designed for *ex vivo* drug screening, involving the evaluation of cell death through immunofluorescence staining using specific antibodies for tumor cell detection and apoptosis over a 10-day period. The protocol employs *ex vivo* cultures, confocal semi high-throughput imaging, and semi-automated image analysis. This setup allows for the simultaneous testing of various compounds and combinations across multiple patient samples. The current application, exemplified for ovarian cancer, has the potential to be extended to other cancer types and drugs through modifications in the antibodies used for tumor cell detection and adjustments in the culture medium. In our current protocol, we utilized only the cleaved caspase 3 antibody to detect cell apoptosis, as it represents the major cell death pathway induced by the compounds tested. However, by incorporating additional antibodies, we can broaden our scope to measure other cell death programs. To assess drug efficacy, tumor cell death is quantified following 48 h of treatment, a duration that can be altered depending on the mechanism of action of the different drugs tested. Importantly, if a specific drug intervention has been recommended for a patient based on genomic background (e.g., *BRCA* mutation or *PIK3CA* mutation), our methods can further test the response to the recommended treatment, bridging the bioinformatic results in an *ex vivo* setup.^9^

## Quantification and statistical analysis

After running the QuPath code “Image_workflow_.groovy” for image analysis, the “ExportMeasurements” command generates the .xlsx file as shown in the example Table 1. The “Marker intensity” is the variable that reflects the total fluorescence of the DAPI, E-Cadherin and cCASP3. The percentage of viable tumor cells (E-Cadherin+) and apoptotic tumor cells (E-cadherin+/cCASP3+) is normalized to the *ex vivo* culture control for the evaluation of on-target drug responses in different treatments. Normalization and statistical evaluation are performed using in-house developed scripts, available from the DRUGSENS r package and *p*-values are calculated through Wilcoxon rank-sum test to compare the relative expression of a cell marker in comparison to the control condition marker.

**Table 1:** Example of raw data exported from DRUGSENS R-package

	filter_image	PID	Date	DOC	Tissue	Image_number	Treatment	Concentration1	Concentration2	ConcentrationUnits1	ConcentrationUnits2	ReplicaOrNot	Treatment_complete	marker_positivity	marker_positivity_ratio
1	PID.001_2023-11-25_14.12.2020_Ascites_series 01_Control_NA_NA_NA_NA_NA_Control	PID.001	25.11.23	14.12.20	Ascites	series 01	Control	NA	NA	NA	NA	NA	Control	E-Cadherin_ratio_of_total_cells	0.94
2	PID.001_2023-11-25_14.12.2020_Ascites_series 01_Control_NA_NA_NA_NA_NA_Control	PID.001	25.11.23	14.12.20	Ascites	series 01	Control	NA	NA	NA	NA	NA	Control	E-Cadherin and cCASP3_ratio_of_total_cells	0.05
3	PID.001_2023-11-25_14.12.2020_Ascites_series 01_Control_NA_NA_NA_NA_NA_Control	PID.001	25.11.23	14.12.20	Ascites	series 01	Control	NA	NA	NA	NA	NA	Control	cCASP3_ratio_of_total_cells	0
4	PID.001_2023-11-25_14.12.2020_Ascites_series 01_Control_NA_NA_NA_NA_NA_Control	PID.001	25.11.23	14.12.20	Ascites	series 01	Control	NA	NA	NA	NA	NA	Control	onlyDAPIPositve_ratio_of_total_cells	0.02
5	PID.001_2023-11-25_14.12.2020_Ascites_series 01_Dmso_NA_NA_NA_NA_NA_Dmso	PID.001	25.11.23	14.12.20	Ascites	series 01	Dmso	NA	NA	NA	NA	NA	Dmso	E-Cadherin_ratio_of_total_cells	0.68
6	PID.001_2023-11-25_14.12.2020_Ascites_series 01_Dmso_NA_NA_NA_NA_NA_Dmso	PID.001	25.11.23	14.12.20	Ascites	series 01	Dmso	NA	NA	NA	NA	NA	Dmso	E-Cadherin and cCASP3_ratio_of_total_cells	0.05
7	PID.001_2023-11-25_14.12.2020_Ascites_series 01_Dmso_NA_NA_NA_NA_NA_Dmso	PID.001	25.11.23	14.12.20	Ascites	series 01	Dmso	NA	NA	NA	NA	NA	Dmso	cCASP3_ratio_of_total_cells	0
8	PID.001_2023-11-25_14.12.2020_Ascites_series 01_Dmso_NA_NA_NA_NA_NA_Dmso	PID.001	25.11.23	14.12.20	Ascites	series 01	Dmso	NA	NA	NA	NA	NA	Dmso	onlyDAPIPositve_ratio_of_total_cells	0.28
9	PID.001_2023-11-25_14.12.2020_Ascites_series 01_Olaparib_10_NA_uM_NA_NA_Olaparib10uM	PID.001	25.11.23	14.12.20	Ascites	series 01	Olaparib	10	NA	uM	NA	NA	Olaparib10uM	E-Cadherin_ratio_of_total_cells	0.77
10	PID.001_2023-11-25_14.12.2020_Ascites_series 01_Olaparib_10_NA_uM_NA_NA_Olaparib10uM	PID.001	25.11.23	14.12.20	Ascites	series 01	Olaparib	10	NA	uM	NA	NA	Olaparib10uM	E-Cadherin and cCASP3_ratio_of_total_cells	0.21

## Limitations

One potential limitation of this protocol lies in the large number of cells (250,000 cells) required to test one condition. This substantial number of cells places constraints on the scope of drug testing, limiting the number of drugs or drug combinations that can be evaluated. As a result, the protocol’s capacity to comprehensively explore a wide array of potential treatments or interventions is limited, which restricts high throughput strategies.

The efficacy of the presented protocol relies on the specificity and reliability of the selected antibodies. Shortcomings or inaccuracies in antibody specificity could impact the precision and validity of the results obtained. Therefore, it is essential to adopt and thoroughly test markers and culture conditions when applied to different subtypes of EOC or other cancer entities.

## Troubleshooting

### Problem 1 (preparation 1e)

The 3D Petri Dish micro-chip can be damaged if they are carelessly removed from their mold.

### Potential solution

Make sure to proceed with care while removing the 3D Petri Dish micro-chip from the mold. Gently loosen each side of the chip entirely before gently popping it out from the center.

### Problem 2 (major steps 1c and 2j)

The cell pellet from either fresh ascites, frozen ascites, or tissue appears red in color.

### Potential solution

You can perform the lysis of erythrocytes by resuspending the pellet in 10 mL of 1× RBC lysis buffer. Vortex for 5 s. Incubate for 2 min at 20°C–22°C and centrifuge at 300 × *g* for 5 min and remove the supernatant.

### Problem 3 (major steps 1c and 2j)

Substantial cell debris (>40%).

### Potential solution

You can execute the debris removal process as follows: resuspend the cell suspension in 6200 μL of cold PBS (4°C) (avoid vortexing), then add 1800 μL of debris removal solution. Thoroughly mix by pipetting slowly up and down ten times using a 5 mL pipette. Overlay the mixture with 4 mL of cold PBS (4°C) and centrifuge at 3000 × *g* for 10 min at 4°C, employing full acceleration and full brake to ensure the formation of three phases. Discard the top two phases entirely and add cold PBS (4°C) to achieve a final volume of 15 mL. Invert the tube three times (avoid vortexing), centrifuge at 4°C, 1000 × *g* for 10 min with full acceleration and full brake, and remove the supernatant completely.

### Problem 4 (major step 1d)

Cell viability below 50%.

### Potential solution

If the cell viability is below 50% you can execute the dead cell removal process as outlined below: for every 10^7^ total cells, dilute 0.25 mL of 20× Binding Buffer Stock Solution with 4.75 mL of sterile, distilled water. Centrifuge the cells at 300 × *g* for 5 min at 20°C–22°C and remove the supernatant. Resuspend the pellet in 100 μL of Dead Cell Removal MicroBeads for approximately 10^7^ total cells, ensuring a thorough mix, and incubate for 15 min at 20°C–22°C. Choose an LS positive column type, place the column in the magnetic field of a suitable QuadroMACS Separator, and activate the column by flushing with 3 mL of 1× Binding Buffer. Apply the cell suspension onto the column, allowing live cells to pass through. Rinse the column four times with 3 mL of 1× Binding Buffer, collecting the effluent as the live cells fraction. Centrifuge the cells at 300 × *g* for 5 min at 20°C–22°C and completely remove the supernatant.

### Problem 5 (major step 9d)

A lack of precision during the sectioning process can cause paraffin blocks to break, leading to sample loss. This setback may require a second experiment, causing significant time and resource burdens.

### Potential solution

Special care is needed, particularly during the trimming process. Prior to switching to auto mode, manually test the sectioning machine every time. Once you reach the correct level of your paraffin block, confirm the presence of spheroids under the microscope.

## Resource availability

### Lead contact

Further information and requests for resources and reagents should be directed to and will be fulfilled by the lead contact, Francis Jacob (francis.jacob@unibas.ch).

### Technical contact

Francis Jacob (francis.jacob@unibas.ch).

### Materials availability

All materials used in this experiment are available through commercial resources.

### Data and code availability

To the best of our knowledge, we have provided all information necessary. If any further information is required to perform the experiment described in this work, the lead contact welcomes requests.

The workflow can be reproduced by a custom-made package developed ad-hoc for QuPath-based data. The package can be found in GitLab repository: https://git.scicore.unibas.ch/ovca-research/drugsens and its forward mirror to GitHub repository: https://github.com/flalom/drugsens. An example workflow and the package’s instructions are shown in the repositories.
